# Pulsed Intra-Arterial Drug Injection during Diastolic Phase of Cardiac Function Increases Drug Efficacy by Enhancing Pharmacological Exposure of Targeted Tissues

**Published:** 2016-06-01

**Authors:** M. Rismanchi

**Affiliations:** 1 Department of Neurology, Shiraz University of Medical Sciences,Shiraz, Iran

**Keywords:** Area Under the Curve, Diastolic Phase, Drug Exposure, Intra-Arterial Drug Injection, Pulsatile Injection, t-PA

## Abstract

Diastolic phase of cardiac function is associated with lower arterial flow and hence higher concentration of intra arterially injected drug is achieved at the site of injection. It is herein postulated that drugs show higher efficacy when injected during the diastolic phase of cardiac function. It is also postulated that this benefit cannot be achieved when the drug is injected with higher rates thus producing the same high concentration at the site of injection. Pulsed intra arterial injection also benefits from the delayed therapeutic effect of the decaying drug before the next shot of injection resaturates the targeted tissue. Altogether, it is estimated that diastolic time-locked pulsed intra arterial injection will increase the drug efficacy up to 1.9 times the efficacy of injected drug with conventional methods. This is significant for drugs with limited dose of administration due to their disastrous side effects like tissue plasminogen activator or chemotherapeutic drugs.

## Introduction

Pulsed intra-arterial drug injection during diastolic phase of cardiac function increases drug efficacy by enhancing pharmacological exposure of targeted tissues. Intra direct drug exposure by intra arterial injection has therapeutic effects attributable to the concentration of the drug at the site of action and the duration that the target tissue is exposed to the drug. Examples include intra-arterial tissue plasminogen activator (t-PA) injection in an occluded artery or intra-arterial chemotherapeutic drug injection in the supplying arteries of a tumor. Analyzing t-PA as an example, the real exposure of clot to the activated plasmin can be studied by non-compartmental analysis in the field of pharmacokinetics[1]. The degree of exposure following administration of a drug is described by area under the curve (AUC) calculated from plasma drug concentration versus time curve[2]. Regarding this factor, increasing the rate of drug injection, though increasing drug concentration, decreases exposure time to the drug. Altogether, it does not affect the AUC though increasing the local concentration of the injected drug, but intermittent intra-arterial injection does increase the AUC.

The concentration of a chemical is determined through equation 1:

$$C_{0}=\frac{mg}{V}\;\;(1)$$

In which *C_0_*, *mg* and *V* indicate concentration, milligram of drug and volume of fluid. While in a flowing fluid, Volume of diluting blood (V) is also determined by the hemodynamic rules, equations 2 and 3:

V=Qt  (2)

$$Q=\frac{\pi r^{4}}{8\eta L}\times P\;\;(3)$$

Where *Q*, *r*,* L* and *P* stand for flow rate, arterial radius, length and η is fluid viscosity and π is the mathematical constant Pi. Assuming that the body vasculature is arranged in parallel pattern with P accounting for pressure difference between arterial and venous ends of each vascular units, here it is roughly estimated that P is equal to blood pressure. Substituting equations 2 and 3 for equation 1 and solving it for resultant concentration ratio, it yields:

$$\frac{C}{C_{0}}=\frac{P_{0}}{P}\;\;(4)$$

This formula indicates the inverse dependence of local concentration of the injected t-PA to the blood pressure (*P*) at the site of injection. Furthermore, the activated plasmin in the targeted clot obeys the decaying rules in pharmacokinetics and is determined by plasmin half-life (* T_0.5_*) based on the following equation:

$$\int C=-\frac{T_{0.5}\times C_{0}}{\ln2}\times 2^{-\left(\frac{t}{T_{0.5}}\right)}\;\;\;(5)$$

**Figure 1:**
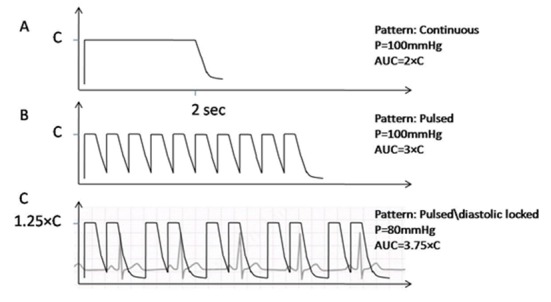
A. intra-arterial drug injection during 2 sec period. B. Intra-arterial drug injection with 10 shots of injection with duration of 0.2 sec and intervals of 0.2 sec corresponding to the 2nd half-life of plasmin. C. Pulsed injection of t-PA during diastolic phase of cardiac function both increases the drug concentration with a factor of 1.25 and AUC with a factor of 3.75. C, concentration; P, blood pressure; AUC, area under the concentration versus time curve.

*T_0.5_* accounts for plasmin half life that is equal to 0.1 sec[3] and C accounts for plasmin concentration at time t. Calculating *AUC_0_* for 2ml (concentration of C) intra-arterial injection of t-PA during 2sec (figure 1a) and comparing with *AUC_1_* calculated when the drug is administered intermittently in 10 shots and within 0.2 sec intervals (0.2 sec accounts for 2^nd^ half-life of plasmin) (figure 1b), it yields *AUC_1_*:* AUC_0_* ratio of 1.5. This measurement is estimated when drug delivery is performed with no regards to the cardiac systolic function assuming that P is equal to mean arterial pressure (MAP) of 100 mmHg. Performing the procedure during the diastolic phase of cardiac function with P equating 80 mmHg, the ratio of *AUC_2_*:* AUC_0_* becomes 1.875 (figure 1c). According to these proves, t-PA administration during diastolic phase of cardiac function with a pattern of pulsed injection that consists of intervals equal to 2^nd^ half-life of plasmin, 30 mg t-PA has the efficiency of injecting 56.25 mg of the drug when injected through conventional methods. It is a matter of further research whether the side effects also increase with this method. The proposed effect for pulsed intra-arterial injection of t-PA is also compatible with the administration of other drugs like Streptokinase and chemotherapeutic drugs. The area of drug administration through therapeutic methods of delivery is promising for increasing the final therapeutic outcome and especially has to be considered as a new technique used in recently equivocal trials of early stroke treatment by intra-arterial t-PA[4, 5].
